# 3D exoprosthesis in socket model for dog with amputed pelvic limb: case report

**DOI:** 10.1186/s12917-025-04574-6

**Published:** 2025-02-26

**Authors:** Miriã Mamede Noronha de Souza, Marciana da Cunha Antonioli, Matheus Henrique Martins dos Santos, Bianca Medeiros dos Santos Véras, Lucas Rannier Ribeiro Antonino Carvalho

**Affiliations:** 1 Postgraduate Program in Veterinary Medicine, Center of Health and Rural Technology (CSTR), Federal University of Campina Grande (UFCG), Patos, Brazil; 2 Department of Veterinary Medicine, University Center of João Pessoa - UNIPÊ, João Pessoa, Brazil; 3 Department of Physiology and Pharmacology, Karolinska Institutet, Solnavägen 9, Stockholm, S-171 77 Sweden

**Keywords:** Well-being, Dogs, 3D medicine, Biomechanics, Prosthesis, PLA

## Abstract

**Background:**

Disorders of the locomotor system in dogs, such as amputations or malformations, can be not only physically but also emotionally distressing. In this context, advances in medical and technological sciences offer tools and options with the aim of improving the quality of life of animals with locomotor problems. This case report aims to describe the custom development of a 3D exoprosthesis for a dog with an amputated hind limb.

**Case presentation:**

A female dog, mixed breed, approximately 5 years old, was admitted to the Veterinary Clinic of the Centro Universitário de João Pessoa—Brazil, with locomotor problems due to low amputation of the left hind limb, without pain or sensitivity to touch on the amputated stump. Measurements of the amputated limb were collected to create a virtual model of the 3D exoprosthesis in a socket model. After simulations and tests, the prosthesis was materialized by 3D printing in collaboration with the Brazilian company 3D Medicine©, using polylactic acid (PLA) as the main material, an organic, lightweight, and resistant synthetic thermoplastic. The exoprosthesis was covered with protective material and fixed to the animal with a compressive bandage. Immediately after fixation, the animal demonstrated support of the limb on the prosthesis while standing, better distribution of body weight and relief of load on the contralateral limb. The increase in the time of use of the prosthesis was gradual and under supervision, after four weeks the dog did not present major difficulties in walking, running, and eating, in addition, no injuries to the amputated stump were observed. Veterinary physiotherapeutic follow-up was recommended.

**Conclusion:**

This case report describes the development of a 3D exoprosthesis for dogs as a cost-effective option to reduce the locomotor impacts of limb amputation and improve quality of life. Techniques using additive manufacturing and 3D technology have immense potential for medical application, especially in veterinary medicine due to the difference in anatomical and body structure between domestic and wild animals.

## Background

According to the Brazilian Association of the Pet Products Industry (ABINPET), there are more than 67.8 million dogs in Brazil, which highlights the importance of these animals in the lives of the population and society. These changes in the profile of society and family structure are directly reflected in animal care and veterinary services, with the central objective of ensuring a decent quality of life and well-being [1].

In companion animals, congenital malformations in the limbs and amputations due to physical injuries or surgical procedures are the main causes of locomotor disorders. In fact, limb amputation is one of the oldest surgical techniques documented in humans and animals [2]. For animals, biomechanical changes are directly reflected in quality of life, and the absence of movements and social interactions can dramatically reduce longevity [3].

In specific cases, surgical removal of the limb or part of it aims to preserve the animal’s health and life in the face of serious injuries that can progress to systemic damage such as necrosis (physical injuries and trauma) and metastasis (neoplastic proliferation) [4]. In cases where partial amputation is performed, the use of prostheses may be recommended to reduce the damage caused by the absence of the limb, however, the indication for prosthesis depends on many other factors such as the species, patient temperament and availability of the person responsible for monitoring [5].

Orthopedic prostheses in veterinary medicine can be classified according to the fixation method into: (1) Endoprostheses when surgically fixed to the animal’s bone structures, or (2) exoprostheses when externally fixed [2]. In both cases, the objective is to offer better stability to the movement and reduce the impacts caused by the absence of the limb on the animal’s biomechanics. In this scenario, the prosthetics industry has significantly advanced in the field of veterinary medicine following developments in the human medical sector [6].

In animals, three-dimensional (3D) prosthetics are also divided into: (1) *structural* when used to replace parts of the body such as beaks and shells [7, 8] and (2) *orthopedic*, when used to simulate parts of the limbs, thoracic or pelvic [3, 9, 10]. Prosthetics printed using additive manufacturing or 3D printing enable customized development, according to the individual needs of each animal. This technology, widely used in other areas such as engineering and architecture, was registered in the early 1980s and medical applications were immediately recognized and explored from the 1990s onwards [11].

One of the key points of using 3D technology is to use the patient’s measurements, 3D scanner and/or computerized tomography (CT scan), to create a digital model of the prosthesis with high precision. The file is custom-made based on the anatomical characteristics of the amputated/malformed region and can be materialized by different methods and materials, such as nylon, metal, resins, and diverse types of organic or non-organic plastics [12, 13].

Facing the exciting potential for the use of 3D technology in veterinary medicine and the need for prototypes and custom-designed models for animals, the objective of this report was to describe the development of the 3D exoprosthesis in a socket model for the pelvic limb of a dog that was rescued with an amputated limb and locomotor disorders.

## Case presentation

**Fig. 1:**
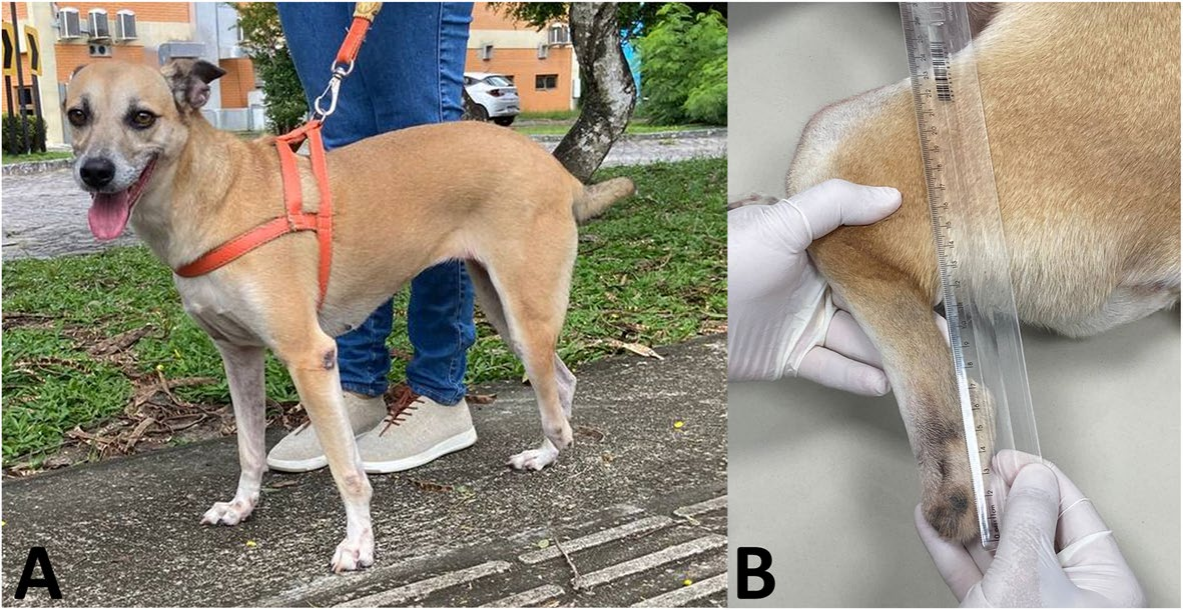
(**A**) Dog, *Canis lupus familiaris*, female, mixed breed, rescued with low amputation of the left hind limb approximately eight centimeters from the ground, (**B**) approximate photographic record of the amputated limb in lateral view

A canine, female, mixed breed, 5 years old, weighing 18.7 kg, was admitted to the Veterinary Clinic of the Centro Universitário de João Pessoa UNIPÊ, with partial amputation of the left pelvic limb in the region of the tarsal joint, at a height of approximately 8 cm from the floor. The animal was abandoned and rescued by a non-profit organization, and for this reason, the cause and time since amputation could not be determined. At the time of the first physical evaluation and complete examination by the veterinary team, the animal presents complete healing of the amputated stump, with no pain on palpation or digital pressure, absence of dermatological lesions due to support in the ground, however, it presents locomotor difficulties, both during ambulation and eating while standing (Fig. 1).

**Fig. 2:**
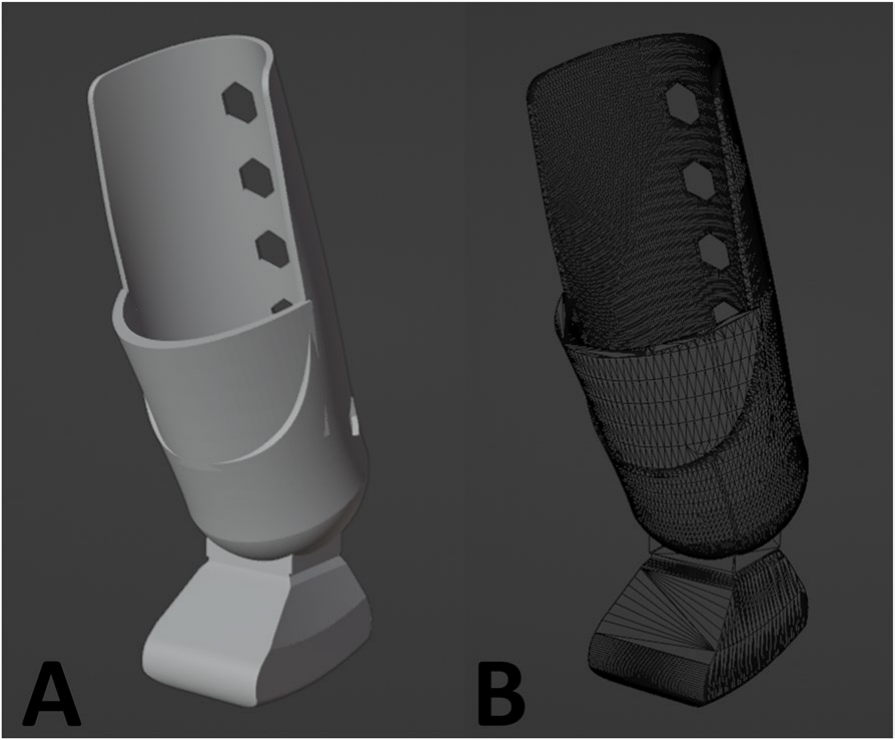
First digital model of the custom-made 3D exoprosthesis measuring 53.4 × 75.6 × 156.8 mm in the 3D modeling software Blender version 3.6, (**A**) solid mode and (**B**) arrayed mode. Photo courtesy: 3D Medicine—@3dmedicine.vet

After routine examinations of the dog, to assess physical integrity and rule out other pathological changes, the use of a 3D exoprosthesis in a socket model with external fixation was recommended, a minimally invasive and cost-effective approach. The digital model of the prosthesis was made based on measurements of the amputated stump of the left limb and using the contralateral intact paw (right limb) as a reference. From these data, the 3D virtual file (Fig. 2) was custom modeled in socket format to fit the amputated stump easily, using the 3D modeling software Blender version 3.6 [14].

During the modeling of the virtual 3D file, simulations were carried out to reproduce the anatomy and angulation of the animal’s paw, as accurately as possible, using the Blender software, with the aim of facilitating the integration of the exoprosthesis with the affected limb and the biomechanics of movement. This modeling resulted in optimized motor and dynamic development, contributing to the animal’s well-being and mobility.

Prosthesis fixation tests were performed using a printed pilot model (Fig. 2) to evaluate the measurements and interaction with the animal. After fitting the test model to the animal’s amputated limb, adjustments were made to improve the development of the final version. In addition, biomechanical tests and simulations were performed, such as tests to assess the height of the femoro-tibio-patellar and tibio-tarsal joints in relation to the contralateral limb and the ground, limb angulation tests with the prosthesis, ambulation tests, limb support and body weight distribution. These physical tests contributed to the identification of areas of weakness in the prosthesis that could require structural reinforcement.

**Fig. 3:**
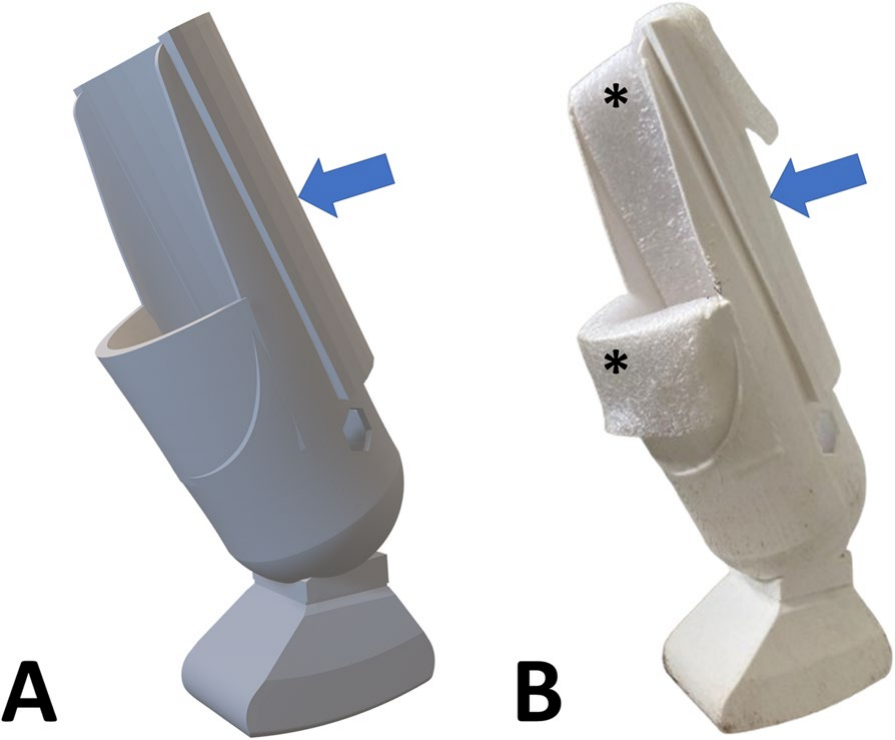
Final version of the 3D exoprosthesis with the addition of the 3.45 mm thick reinforcement wall (blue arrows). (**A**) Virtual model. Photo courtesy: 3D Medicine—@3dmedicine.vet. (**B**) Physical model of the exoprosthesis printed with white PLA filament after removing the printing supports and testing the polyethylene foam layer (4 mm) for internal lining (*)

Using the tests and simulations mentioned above as reference, the final model has a strategically designed fixing cup with reinforcement in areas with higher mechanical tension, and a larger fixing area to avoid stress at focal points (Fig. 3). In addition to a wide and angled base, to provide stability and alignment with the hind limb’s movement pattern, these adaptations were combined with the aim of offering comfortable use during the animal’s movement with the socket prosthesis.

The final model was prepared for 3D printing using the Ultimaker Cura® 5.3.0 software, or “slicer” software, and the model materialized by 3D printing using fused deposition modeling (FDM) technology by Creality CR10 Smart 3D printer (Creality 3D Technology Ltd., Shenzhen, China). Adaptations of the methods described by Carvalho [9] were used, such as a 0.4 mm printing nozzle, 0.2 mm layer height and 70 mm/s printing speed. 3D printing was carried out in partnership with the Brazilian company 3D Medicine©, through the program to support the development of new 3D technologies applied to veterinary medicine.

After 3D printing the prostheses, removing the printing supports, sanding, and finishing the edges was performed, to ensure a smooth surface without areas of abrasion (Fig. 3B). The cup-shaped area was coated with polyethylene foam (4 mm) to improve fixation and prevent injury to the amputated stump and a non-slip shoe sole rubber (6 mm) was added in the area that had contact with the ground, in order to minimize slips and falls during the adaptation process. Both components were adhered to the exoprosthesis using “superglue” based on cyanoacrylate, with medium viscosity and high curing speed (Tekbond Instant Adhesive 793 Multi-Purpose).

**Fig. 4:**
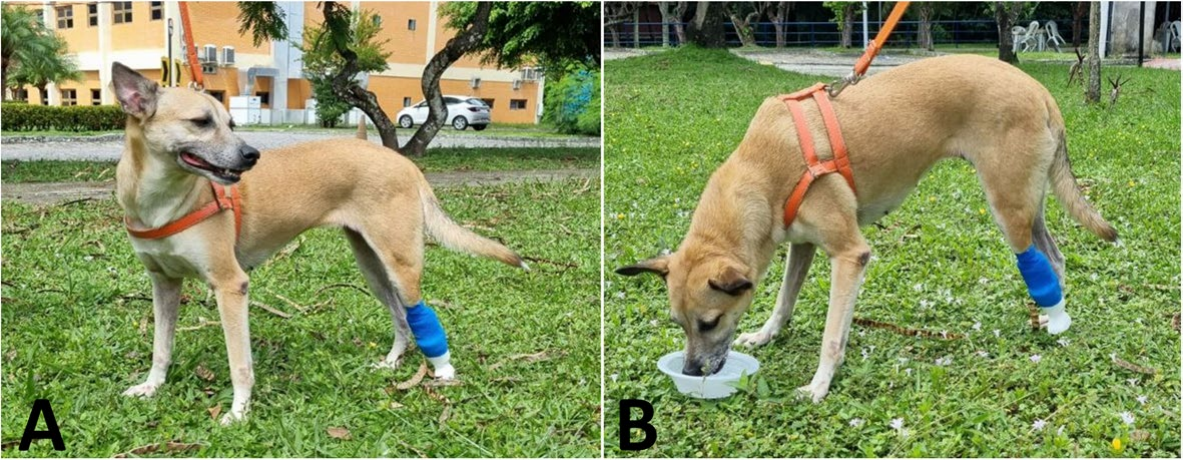
Photographic records of the first contact after fixation of the exoprosthesis. (**A**) Dog in station after short walk with evident support of the amputated limb under the prosthesis and better distribution of body weight and alignment of the joints of the hind limb. (**B**) Animal drinking water with support of the amputated hind limb on the ground and apparent more comfort for daily activities

On the first day of adaptation and tests, the animal’s amputated stump was covered with a regular cotton sock (65% cotton, 32% polyamide and 3% other fiber) and inserted into the C-shaped area of the exoprosthesis. After a comfortable fit, the limb was fixed with compressive bandage. Immediately after fixing the exoprosthesis, the animal demonstrated a better distribution of body weight with the support of the prosthesis on the ground (grassy area chosen for less impact), reducing the load on the contralateral limb (Fig. 4A).

Even after just a few minutes of use, it was possible to identify greater comfort during daily activities, such as locomotion, feeding and interactions with other animals (Fig. 4B). The tutors were informed regarding the need for the prosthesis special care, including daily removal for cleaning and to observe the stump area, in order to avoid possible injuries. In addition to referral to the veterinary physiotherapy service, for instruction and evaluation of the adaptive process. At the end of the first test using the exoprosthesis (1 h), the dog did not show any irritation or abrasive lesions on the skin.

After two weeks of use, the exoprosthesis showed some wear on the rubber coating added to the sole due to friction with the floor, which was replaced with the same material but with a higher thickness (10 mm). No changes or injuries were identified in the skin of the amputated stump. The animal was monitored for four weeks and no complaints from the owner were reported. An evaluation and follow-up for longer periods along with communication with the veterinary physiotherapy service is recommended, however, as this is a rescued animal that was homeless, it was not possible to obtain more information about the management of the case.

## Discussion and conclusions

This report explores new possibilities for manufacturing prosthetic models for companion animals using low-cost 3D technology. In veterinary medicine, there are two main types of limb prostheses: exoprosthesis (prostheses with external fixation) and endo-exo prosthesis (EEP) with internal fixation to the bone [2]. These two models aim to improve biomechanics and reduce the impacts caused by the absence of a limb, the main difference being the way of fixing the amputated limb [4].

Exoprosthesis, also called socket prosthesis, are generally shaped like a padded cup for insertion into the amputated stump and external fixation by bandages, they are used in incomplete limbs with the aim of improving locomotion and reducing impacts on contralateral joints, they are less invasive and do not require surgical procedures for implantation, however, this type of prosthetic models present more difficulties regarding fixation methods [4].

In the other hand, endo-exoprostheses are fixed to the bone end of the amputated stump by a surgical procedure, offering a more stable and resistant fixation point for the prosthesis [15]. These prosthesis models with bone fixation are mainly used when the patient undergoes planned amputations, and during the amputation surgery the prosthesis fixation pin is already inserted, avoiding a new surgical procedure and a combined healing process [16, 17].

For this case report, the dog presented a healed amputated stump without painful sensitivity in the extremity. Based on the results of clinical evaluations, a socket prosthesis was recommended, as it offers benefits such as low cost and high resistance, easier maintenance and cleaning, and mainly due to the possibility of development without undergoing an invasive surgical procedure. The 3D model was custom designed according to the dog’s biomechanical characteristics, morphology and behavioral patterns. A production process similar to that reported by Kastlunger [10], who developed a 3D personalized prosthesis for a 1-year-old German Shepherd dog with congenital deformity in the right forelimb and Carvalho [9] who developed 3D socket prosthetics for small and medium-sized birds.

Regarding the material used, the exoprosthesis was printed with an organic plastic filament, polylactic acid (PLA) (3D Lab Industria Ltd, Belo Horizonte, Brazil), chosen due to the balance between cost–benefit, mechanical characteristics and practicality. This material is one of the most used in 3D printing by FDM due to its lightness, resistance, chemical characteristics and origin from renewable sources, such as corn starch, sugar cane and beet [13]. Furthermore, PLA does not present major risks to the animal’s health if the material is ingested, ensuring greater safety during the use of the exoprosthesis, as it is relatively common for dogs to chew and ingest parts of prostheses and other plastic objects.

The thermoplastic polymers, such as PLA, are suitable for producing 3D models of prosthetic limbs, with ABS (acrylonitrile butadiene styrene) being another viable alternative. Additionally, polyamides are known for their remarkable properties of dimensional stability, rigidity, flexibility, and high impact resistance, making them ideal for applications that require durability and shock absorption [18]. Facing the difficulties in fixing socket prostheses, for this case, the cup-shaped structure of the exoprosthesis was custom designed and internally coated to provide greater comfort and avoid dermal injuries. The model was fixed externally using compressive bandages (VitalTape, São Paulo, Brazil) after insertion of the amputated stump covered with a cotton sock for better protection and comfort, similar to the use of prostheses in humans [10].

The height of the amputation and consequently the height of the prosthesis are also critical points in the prosthetic manufacturing process. In this regard, exoprostheses have a higher success rate and better adaptation in dogs with low limb amputations, that is, distal to the carpal and tarsal joints. Mainly due to the larger area for external fixation of the prosthesis in the structure of the residual limb [5]. Corso [19] describes that, in general terms, the preservation of at least half of the ulna/radius and/or the tibia/fibula provides good fixation of the prosthesis and the immediate use of socket-type prostheses when there is a veterinary indication.

The process of adapting an animal to a prosthesis (whether exo or endoexo) requires a gradual and careful approach, with the possible need for physical adjustments to the prototype, patience and supervision on the part of the owners. Positive stimuli, such as rewards and other positive reinforcement techniques, helped to associate the prosthesis with positive experiences, facilitating adaptation, ensuring comfort, adequate adjustment and constant monitoring in order to avoid discomfort or injuries [5, 18]. In this case report, the dog showed excellent acceptance and adaptation results, through training allowing the ability to walk autonomously and as naturally as possible. The behavioral characteristics of each animal can directly reflect on the adaptation process, in fact, according to Marcellin-Little and collaborators [4] there is a recommended restriction for applying these devices to animals with aggressive behavior. And in these cases, appropriate behavioral monitoring and treatment must be conducted before implanting the prosthesis.

As seen in the report, the use of the 3D exoprosthesis was comfortable for the dog from the first contact, with no signs of pain or injuries. The prosthesis was the correct size to stabilize the dog’s gait and redistribute body weight, without overloading the contralateral joints. In the long term, it would be interesting to perform fatigue, tensile and impact tests to determine failure points and potential use over months. Furthermore, frequent monitoring by owners and veterinarians is necessary to assess musculoskeletal health, gait and biomechanics and identify possible injuries and needs for adjustments in the size of the prosthesis, especially in young animals still growing [20].

This report describes the use of three-dimensional technology for modeling and printing an exoprosthesis for a domestic dog with a direct impact on the animal’s quality of life and well-being. The model was custom developed, printed at low cost with light, resistant and non-toxic material. For veterinary medicine, a technology that offers customized production of products and models is of great value given the anatomical variability of patients and the need for lightweight and adjustable materials [18, 21].

The use of 3D technology in animal health is in constant development, with different possibilities for application such as the manufacture of orthopedic and structural prostheses, virtual or physical surgical planning, production of models for anatomical studies and surgical tools [12]. An advance in parallel with the growing use in other areas such as human health, engineering and industry. However, the applications of 3D modeling and printing technology are still unknown to many animal science professionals and more dissemination of methods, results and case reports in domestic and wild animals is needed.

## Data Availability

No datasets were generated or analysed during the current study.

## Abbreviations

3D: Three-dimensional
FDM: Fused deposition modeling
CT: Computerized tomography
AM: Additive manufacturing
PLA: Polylactic acid
EEP: Endo-exo prosthesis
cm: Centimeter
mm: Millimeter
